# An Insecticide Further Enhances Experience-Dependent Increased Behavioural Responses to Sex Pheromone in a Pest Insect

**DOI:** 10.1371/journal.pone.0167469

**Published:** 2016-11-30

**Authors:** Antoine Abrieux, Amel Mhamdi, Kaouther K. Rabhi, Julie Egon, Stéphane Debernard, Line Duportets, Hélène Tricoire-Leignel, Sylvia Anton, Christophe Gadenne

**Affiliations:** 1 Neuroéthologie-RCIM, INRA-Université d’Angers, Beaucouzé, France; 2 Département d’Ecologie Sensorielle, Institut d’Ecologie et des Sciences de l’Environnement (IEES), Paris, France; 3 Université Paris-Sud, Université Paris-Saclay, Orsay, France; USDA Agricultural Research Service, UNITED STATES

## Abstract

Neonicotinoid insecticides are widely used to protect plants against pest insects, and insecticide residues remaining in the environment affect both target and non-target organisms. Whereas low doses of neonicotinoids have been shown to disturb the behaviour of pollinating insects, recent studies have revealed that a low dose of the neonicotinoid clothianidin can improve behavioural and neuronal sex pheromone responses in a pest insect, the male moth *Agrotis ipsilon*, and thus potentially improve reproduction. As male moth behaviour depends also on its physiological state and previous experience with sensory signals, we wondered if insecticide effects would be dependent on plasticity of olfactory-guided behaviour. We investigated, using wind tunnel experiments, whether a brief pre-exposure to the sex pheromone could enhance the behavioural response to this important signal in the moth *A*. *ipsilon* at different ages (sexually immature and mature males) and after different delays (2 h and 24 h), and if the insecticide clothianidin would interfere with age effects or the potential pre-exposure-effects. Brief pre-exposure to the pheromone induced an age-independent significant increase of sex pheromone responses 24 h later, whereas sex pheromone responses did not increase significantly 2 h after exposure. However, response delays were significantly shorter compared to naïve males already two hours after exposure. Oral treatment with clothianidin increased sex pheromone responses in sexually mature males, confirming previous results, but did not influence responses in young immature males. Males treated with clothianidin after pre-exposure at day 4 responded significantly more to the sex pheromone at day 5 than males treated with clothianidin only and than males pre-exposed only, revealing an additive effect of experience and the insecticide. Plasticity of sensory systems has thus to be taken into account when investigating the effects of sublethal doses of insecticides on behaviour.

## Introduction

The use of large quantities of insecticides in plant protection strategies leads to low dose residues in the environment [1, 2]. The most frequently used insecticides today are neonicotinoids with a half-life ranging from a few days to more than 1000 days [1]. Neonicotinoid insecticides target nicotinic acetylcholine receptors (nAChRs), responsible for synaptic transmission in the central nervous system of insects and playing a major role in sensory systems [3]. Sublethal doses of these neonicotinoids have been shown to disturb behaviour and memory performance in non-target pollinating insects [4–7]. On the other hand, recent studies have discovered that low doses of neonicotinoids can improve reproduction in various target insects, thus showing a hormesis effect [8, 9]. Because male moths have a specialized, highly sensitive olfactory subsystem dedicated to detection and processing of the female-emitted sex pheromone, leading ultimately to an oriented behaviour towards the conspecific pheromone signal [10], moths are ideal pest model organisms to study effects of low doses of neonicotinoids on olfactory-guided behaviour. Indeed synaptic transmission involved in olfaction is mainly cholinergic, and neonicotinoid residues could modify the chemical communication system and consequently affect reproductive success in these pest insects.

In the migratory moth *Agrotis ipsilon*, we have shown that acute oral treatments of sexually mature males with a low dose of clothianidin (10 ng corresponding to the LD20) increased their behavioural responses to the sex pheromone. This hormetic-like effect was found to be correlated with an increase in pheromone sensitivity within the central, but not the peripheral olfactory system [11, 12]. As insect behaviour is highly plastic and depends on physiological state, i.e. sexual maturity, and previous experience of an individual, it is important to study the effects of insecticides in relation to the factors inducing plasticity of behavioural responses.

To cope with variations of their ever-changing environment, insects have developed behavioural plasticity as an adaptive strategy and to increase fitness [13]. This allows individuals to adapt to variations of abiotic conditions and to changes in intra- and interspecific interactions. One strategy to adapt to the environment and increase fitness is to take advantage of earlier sensory experience, i.e. learning [14]. There is now strong evidence for different forms of learning, such as associative learning and configural learning that exist predominantly in social insects, e.g. honeybees, but also in non-social insects such as fruitflies and moths [15–18]. However, non-associative learning such as habituation and sensitization caused by repeated exposure to sensory stimuli has been shown in many species [19, 20]. Sensory experience can affect various behaviours by influencing the function of neural circuits in both vertebrates and invertebrates [21–24].

Regarding reproduction, an accurate and rapid identification of a suitable partner is essential to avoid a waste of time and energy and to decrease the risk for predation, thus suggesting that reproductive signals should be stable and their detection less affected by experience. There is now, however, evidence that prior experience also shapes the perception of reproductive sensory cues. Such a type of olfactory plasticity based on non-associative learning has been unveiled in the moth *Spodoptera littoralis*: a brief pre-exposure to sex pheromone enhances behavioural responses to sex pheromone 15 min and 24 h later [25, 26]. Pheromone exposure was also shown to enhance peripheral pheromone detection, central nervous pheromone responses, and to induce an increase in the volume of certain glomeruli and specifically the size of the biggest glomerulus processing the main pheromone component within the antennal lobe (AL), the primary olfactory centre, as well as in a secondary olfactory centre, the calyces of the mushroom bodies [25, 27, 28]. It is so far unknown if such pre-exposure effects are also present in other insects and if they depend on the physiological state of the insect.

In *A*. *ipsilon*, males are not sexually mature when they hatch from the pupa and reach maturity only after a few days of adult life [29]. As a consequence, responses of males to the sex pheromone are age-dependent: the behavioural response and the neuron sensitivity within the AL to the sex pheromone increase with age [29–31]. We considered thus this species as a suitable model to investigate if pre-exposure to the sex pheromone modifies oriented behaviour as a function of age, and to study if the previously reported unexpected effect of the insecticide clothianidin on pheromone-guided behaviour is dependent on age or previous experience. We used wind tunnel experiments to first test the responses of young immature and older, sexually mature *A*. *ipsilon* males to the sex pheromone at different time intervals after pre-exposure to the pheromone. Then we compared the effects of LD20 clothianidin treatments in males of different ages. Finally, we combined pre-exposure with clothianidin treatments to investigate if the previously revealed effects of the insecticide depend on experience-induced plasticity of the olfactory system.

## Materials and Methods

### Insects

Experiments were performed with adults of *A*. *ipsilon* originating from a laboratory colony in Angers. The colony was based on field catches in southern France and wild insects are introduced each spring. The animals were reared on an artificial diet [32] in individual cups until pupation. Pupae were sexed and males and females were kept separately in an inverted light/dark cycle (16 h light: 8 h dark photoperiod, with scotophase starting at 10 am) at 22°C. Newly emerged adults were removed from the hatching containers every day, and were given access to a 20% sucrose solution *ad libitum*. The day of emergence was considered as day 0.

### Pre-exposure procedure

**Fig 1:**
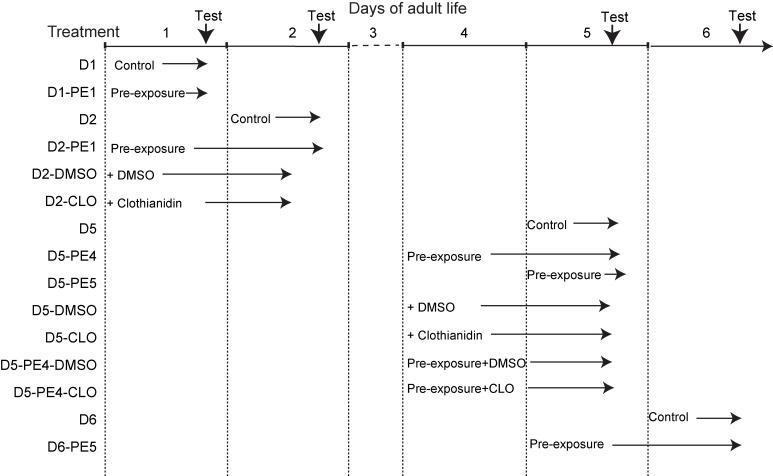
Design of experiments. Treatments were performed on 1-day-, 4-day-, and 5-day-old males, and behavioral tests in wind tunnel were performed either the same day of treatment or 24 h later. Test: behavioral test in wind tunnel.

All pre-exposure treatments were performed in the wind tunnel at mid-scotophase under the same light and temperature conditions as for behavioural pheromone response tests (see next paragraph). Males of the chosen ages were transferred from the rearing room to the wind tunnel room, and placed in plastic cages (maximum of 3 males per cage) before the end of the photophase. A cage was introduced in the wind tunnel 4–5 h after lights off. After 30 s during which the males adjusted to the airflow, a filter paper containing the stimulus was placed 160 cm upwind from the cage. Pheromone stimulation was performed with an artificial pheromone blend containing (Z)-7-dodecen-1-yl acetate (Z7–12:OAc), (Z)-9-tetradecen-1-yl acetate (Z9–14:OAc), and (Z)-11-hexadecen- 1-yl acetate (Z11–16:OAc) (Sigma Aldrich, Saint-Quentin Fallavier, France) at a ratio of 4:1:4 [33, 34]. For pre-exposure, we used a dose of 10 ng pheromone blend, which was previously found to elicit the optimum behavioural response [35]. Each male, kept within the cage, was submitted to pheromone stimulation during 3 min, after which it was removed from the wind tunnel and transferred or not back to the rearing cabinet. Immature (1 day-old) and mature (4 day- and 5 day-old) *A*. *ipsilon* males were pre-exposed, and then tested for behavioural pheromone response either the same day 2 h after the pre-exposure treatment or 24 h later (during the next scotophase) (Fig 1).

### Clothianidin treatments

A clothianidin (99% purity, Sigma-Aldrich, Saint-Quentin Fallavier, France) solution containing 10 ng of clothianidin in 10 μl of the solvent dimethyl sulfoxide (DMSO) was prepared as previously described [12]. The clothianidin dose of 10 ng, which corresponds to the LD20, was chosen because this dose elicited an increase in behavioural and central nervous pheromone responses in 5 day-old *A*. *ipsilon* males, treated at day 4 [11, 12]. To study age-dependence of clothianidin treatments, 1 day-old and 4 day-old males were fed 10 μl of the clothianidin solution or the corresponding DMSO solution as a control and submitted to behavioural tests 24 h later (Fig 1). To combine pre-exposure and clothianidin treatments, 4 day-old males were first pre-exposed in the wind tunnel and then, after 1 to 2 h, fed the clothianidin solution under red light, to be tested at day 5 in the wind tunnel (Fig 1).

### Wind tunnel experiments

Behavioural tests were performed using a 2 m-long flight tunnel under red light illumination as previously described [36]. Experiments were performed during the middle of the scotophase (4–7 h after lights off) when males respond maximally to the sex pheromone [35]. Environmental conditions during the bioassay were held constant: 22°C, 50% relative humidity, wind speed of 0.3 ms^–1^. A cage containing a single experimental male was introduced in the wind tunnel. For pheromone stimulation, the same procedure as described above for pre-exposure was used, except that the dose was this time a suboptimum dose of 1 ng pheromone blend [35]. Only for tests with young males (day 2), which are less sensitive to the sex pheromone, 10 ng of the pheromone blend were used in order to obtain sufficient responses. The behaviour of the moths was observed for 3 min, and partial flight (half of the distance between the source and the cage), complete flight (within 2 cm of the source) and landing on the pheromone source were considered as an oriented response towards the pheromone. We also noted the latency of each oriented response. All experiments were performed double-blind to avoid partial observations. Each day of experiments, different groups of males were tested including at least one group of males that were expected to show a high response level to avoid experimental bias.

For the pre-exposure effect, the following 9 groups of males were tested for their pheromone response in the wind tunnel: 1 day-old pre-exposed at day-1 (D1-PE1) and control males (D1); 2 day-old pre-exposed at day-1 (D2-PE1) and 2 day-old control males (D2); 5 day-old pre-exposed at day-4 (D5-PE4), 5 day-old pre-exposed at day-5 (D5-PE5), and control males (D5); 6 day-old pre-exposed at day-5 (D6-PE5) and 6 day-old control males (D6) (Fig 1).

For the clothianidin effect, the following 6 groups of males were tested: 2 day-old DMSO- and clothianidin-treated at day-1 males (D2-DMSO and D2-CLO respectively) and control males (D2); 5 day-old DMSO- and clothianidin-treated at day-4 males (D5-DMSO and D5-CLO respectively) and control males (D5) (Fig 1).

For the combination of pre-exposure and clothianidin treatments, the following 6 groups of males were tested: 5 day-old DMSO-treated (D5-DMSO), CLO-treated (D5-CLO), pre-exposed at day-4 males (D5-PE4) and control males (D5), as well as males pre-exposed at day 4 and treated with DMSO (D5-PE4-DMSO) and 5 day-old pre-exposed and CLO-treated at day-4 males (D5-PE4-CLO) (Fig 1).

Control groups were repeated for each type of experiments, because response rates can vary throughout the year.

### Statistical analysis

Statistical differences in the percentage of responses between groups of experimental males were evaluated using a R X C test of independence by means of a G-test and applying the Williams’s correction [37]. Delays in responses of males that showed a positive oriented response were compared using the non parametric Kruskal-Wallis test followed by Mann-Whitney tests for pairwise comparisons (P < 0.05) with GraphPad Prism version 6 (GraphPad Sofware).

## Results

### Age-independent effects of pre-exposure

**Fig 2:**
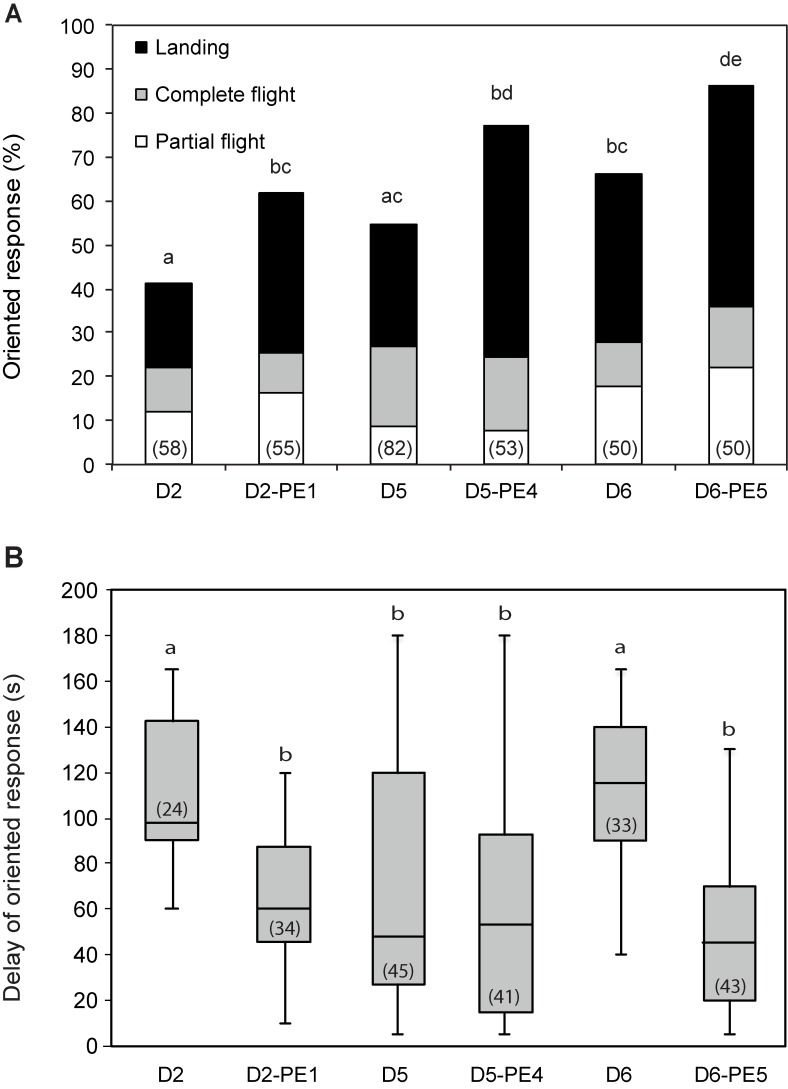
Effect of pheromone pre-exposure on pheromone responses of *A*. *ipsilon* males of different ages tested 24 h after treatment. One-day-, 4-day- and 5-day-old males were pre-exposed for 3 min with 10 ng of the pheromone blend, and their oriented response to a 1 ng pheromone blend was analyzed in wind tunnel experiments 24 h later (D2-PE1, D5-PE4 and D6-PE5 respectively). Unexperienced 2-day-, 5-day- and 6-day-old males (D2, D5, and D6 males respectively) were used as controls. **(A)** Percentage of males showing oriented response. **(B)** Response delay and standard deviation of males showing oriented responses. The lower whisker presents the minimum, the lower hinge of the box is the first quartile, the line inside the box is the median, the upper hinge is the third quartile, and the extreme of the upper whisker is the maximum. Oriented pheromone responses are increased 24 h after brief pre-exposure independently of age. Numbers in bars indicate the numbers of males tested (A) and the number of males that showed an oriented response (B). Bars with the same letter are not significantly different (G-test for (A), Mann-Whitney test for (B), P < 0.05).

The behavioural oriented response of control males significantly increased from day-2 to day-6 (G = 6.66; df = 2; p = 0.035), confirming our previous results [29, 38]. The behavioural response of males that had been pre-exposed increased 24 h later independently of the age (Fig 2). The percentage of males that were pre-exposed, and then tested the following day performing an oriented response was significantly higher as compared with that of D2, D5 and D6 control males respectively (G = 4.15; df = 1; p = 0.041; G = 7.20; df = 1; p = 0.007; and G = 5.53; df = 1; p = 0.018 for D2-PE1, D5-PE4, and D6-PE5 males respectively) (Fig 2A). Detailed analysis shows that there is no statistical difference in the proportions of the 3 different behavioural sequences of the oriented response (partial flight, complete flight, and landing) between the six groups of insects (G = 12.34; df = 10; p = 0.266) (Fig 2A). Response delays of D2-PE1 and D6-PE5 males were significantly shorter than that of D2 and D6 control males (U = 705; p < 0.0001, and U = 1313; p < 0.0001 respectively), but there was no difference in response delays between D5 and D5-PE4 males (U = 1004.5; p = 0.48) (Fig 2B).

**Fig 3:**
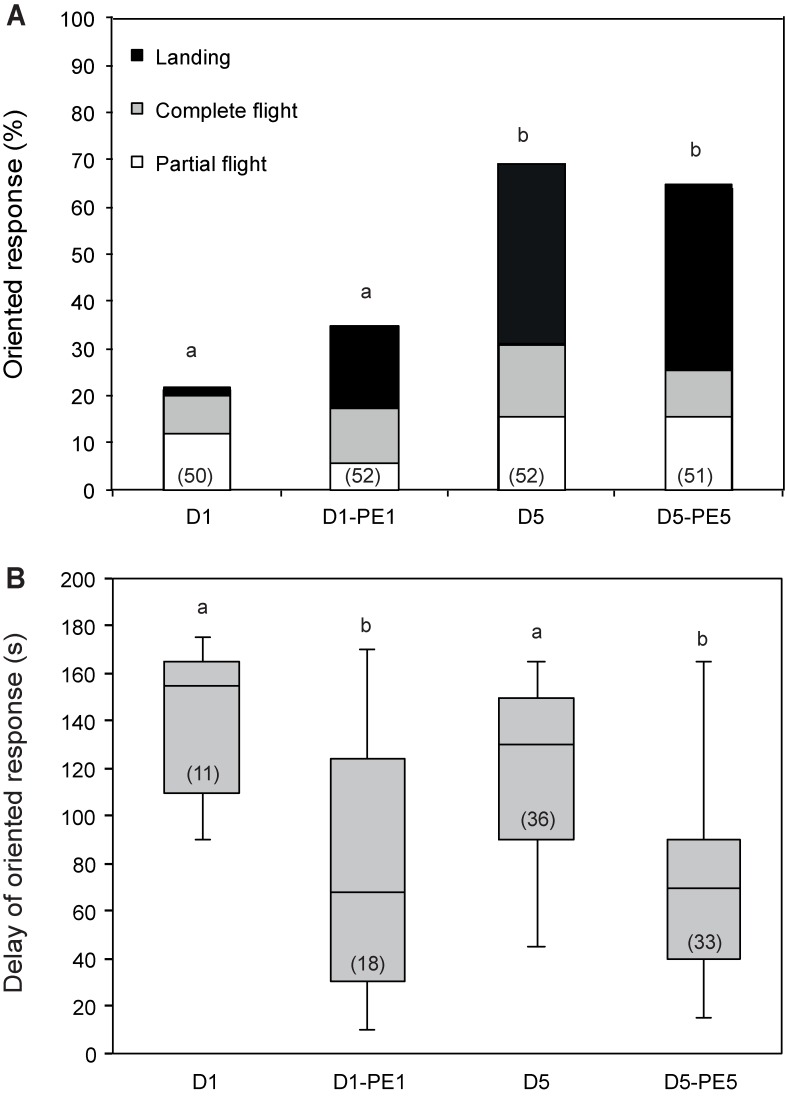
Effect of pheromone pre-exposure on pheromone responses of *A*. *ipsilon* males of different ages tested the same day of treatment. One-day- and 5-day-old males were pre-exposed for 3 min with 10 ng of the pheromone blend, and their oriented response to a 1-ng pheromone blend was analyzed in wind tunnel experiments between 1 and 2 h later (D1-PE1 and D5-PE5 respectively). Unexperienced 1-day- and 5-day-old males (D1 and D5 males respectively) were used as controls. **(A)** Percentage of males showing oriented response. **(B)** Response delay and standard deviation of males showing oriented responses. Oriented pheromone responses of *A*. *ipsilon* males do not increase significantly, but occur with shorter delays less than 2 h after brief pre-exposure independently of age. The lower whisker presents the minimum, the lower hinge of the box is the first quartile, the line inside the box is the median, the upper hinge is the third quartile, and the extreme of the upper whisker is the maximum. Numbers in bars indicate the numbers of males tested (A) and the number of males that showed an oriented response (B). Bars with the same letter are not significantly different (G-test for (A), Mann-Whitney test for (B), P < 0.05).

For the youngest and oldest age groups, responses following pre-exposure were also tested on the same day. There was no significant difference in the percentage of oriented responses of males that were pre-exposed and then tested the same day as compared with D1 and D5 control males (G = 1.98; df = 1; p = 0.159, and G = 0.23; df = 1; p = 0.62 for D1-PE1 and D5-PE5 males tested respectively) (Fig 3A). Detailed analysis shows that there is no statistical difference in the proportions of the 3 different behavioural sequences of the oriented response (partial flight, complete flight, and landing) between the four groups of insects (G = 12.56; df = 6; p = 0.0505) (Fig 3A). However, the response delay of D1-PE1 and D5-PE5 was significantly shorter than that of D1 and D5 control males (U = 164; p = 0.004 and U = 974; p < 0.0001 respectively) (Fig 3B). Thus there is a short and long term pre-exposure effect on the behavioural response delay to the sex pheromone, whereas we only found a long term effect on the percentage of *A*. *ipsilon* males orienting towards the pheromone.

### Age-dependent effects of clothianidin

**Fig 4:**
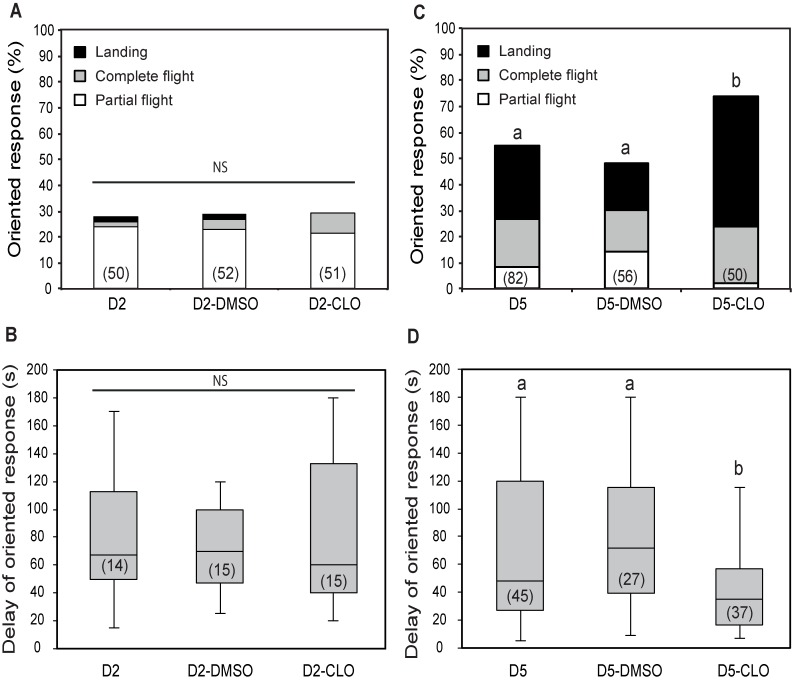
Effect of clothianidin treatment on pheromone responses of *A*. *ipsilon* males of different ages. One-day- and 4-day-old males were orally treated with 10 ng of clothianidin and their oriented response to a 10 and 1 ng pheromone blend was analyzed in wind tunnel experiments 24 h later (D2-CLO and D5-CLO respectively). Unexperienced 2-day- and 5-day-old males (non treated D2 and D5 and DMSO-treated D2-DMSO and D5-DMSO males respectively) were used as controls. **(A, C)** Percentage of 2-day-old and 5-day-old males respectively showing oriented response. **(B, D)** Response delay and standard deviation of 2-day-old and 5-day-old males respectively showing oriented responses. The lower whisker presents the minimum, the lower hinge of the box is the first quartile, the line inside the box is the median, the upper hinge is the third quartile, and the extreme of the upper whisker is the maximum. Oriented pheromone responses improve after clothianidin treatment in 5-day-old, but not in 2-day-old *A*. *ipsilon* males. Numbers in bars indicate the numbers of males tested (A, C) and the number of males that showed an oriented response (B, D). Bars with the same letter are not significantly different (G-test for (A, C), Mann-Whitney test for (B, D), P < 0.05).

Two day-old males treated with 10 ng of clothianidin at day 1 did not change their response behaviour to the sex pheromone as compared to untreated and DMSO-treated males respectively (G = 0.024; df = 2; p = 0.98) (Fig 4A). The distribution of the behavioural sequences could not be statistically analyzed due to the low numbers or absence of complete flight and landing (Fig 4A). Response delays (U = 0.033; p = 0.98) did not change significantly in young males after clothianidin treatment (Fig 4B). On the contrary, the oriented response to the sex pheromone of males at day 5 increased significantly after treatment with the same dose of clothianidin at day 4 as compared to untreated and DMSO-treated males, respectively (G = 4.89; df = 1; p = 0.026; G = 7.37; df = 1; p = 0.0066 for D5/D5-CLO and D5-DMSO/D5-CLO respectively), confirming data obtained in an earlier study (Fig 4C) [12]. Also the distribution of the different sequences showed a significant difference between D5 and D5-CLO (G = 4.93; df = 1; p = 0.026) and between D5-DMSO and D5-CLO (G = 11.3; df = 1; p = 0.0007), and response delays decreased significantly (Fig 4C and 4D). Thus the 10 ng clothianidin dose does not have the same effect on male moths of different ages.

### Clothianidin enhances pre-exposure effects in mature males

**Fig 5:**
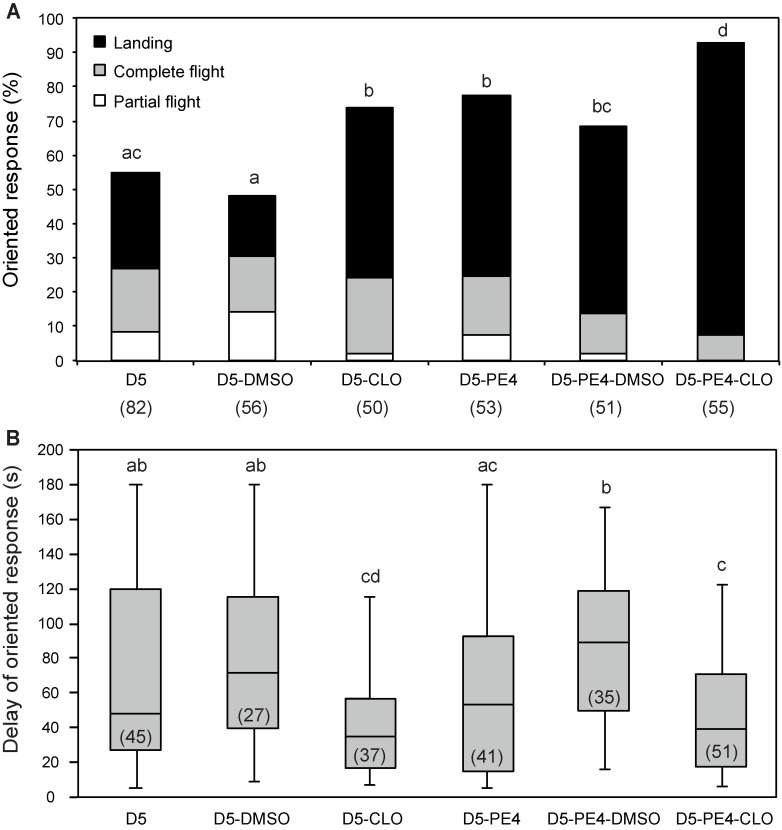
Effect of the combined treatment of pre-exposure and clothianidin on pheromone responses of 5-day-old *A*. *ipsilon* males. Four-day-old males were pre-exposed with 10 ng of the pheromone blend and subsequently orally treated with 10 ng of clothianidin. Behavioural responses to 1 ng of the pheromone blend were observed in the wind tunnel 24 h later (D5-PE4-CLO). Unexperienced and solvent-treated males, as well as pre-exposure- or clothianidin-treated males were used as controls. **(A)** Percentage of males showing oriented response. **(B)** Response delay and standard deviation of males showing oriented responses. The lower whisker presents the minimum, the lower hinge of the box is the first quartile, the line inside the box is the median, the upper hinge is the third quartile, and the extreme of the upper whisker is the maximum. Clothianidin further enhances oriented pheromone responses of *A*. *ipsilon* males after brief pre-exposure. Numbers in bars indicate the numbers of males tested (A) and number of males that showed an oriented response (B). Bars with the same letter are not significantly different (G-test for (A), Mann-Whitney test for (B), P < 0.05).

When combining pre-exposure with clothianidin treatments in sexually mature males, we observed a significant increase of responses to the sex pheromone as compared to either individual treatment (G = 5.16; df = 1; p = 0.023 for D5-PE4-CLO versus D5-PE4; G = 6.91; df = 1; p = 0.008 for D5-PE4-CLO versus D5-CLO) (Fig 5A). In addition, the proportion of males landing on the pheromone source was significantly higher for pre-exposed males treated with clothianidin as compared with males treated with clothianidin only (G = 8.78; df = 1; p = 0.033) and with males pre-exposed only (G = 8.79; df = 1; p = 0.031) (Fig 5A). However the response delay of D5-PE4-CLO was not statistically different from that of single treatments (U = 916; p = 0.819 and U = 1160; p = 0.37 for D5-CLO/D5-PE4-CLO and D5-PE4/D5-PE4-CLO respectively) (Fig 5B).

## Discussion

In this study, we show that brief pre-exposure to the sex pheromone increases pheromone responses in *A*. *ipsilon* males in a similar way at different ages, whereas the insecticide clothianidin at a 10 ng dose only enhances behaviour in sexually mature males. Pre-exposure and clothianidin treatment applied together lead to a further increased behavioural response both in terms of a higher percentage of responding males and a reduced response delay.

The results in *A*. *ipsilon*, revealing a positive pheromone pre-exposure effect (enhancement of pheromone response) confirm previous results obtained in *S*. *littoralis* [25, 26], and extend the findings to sexually immature males. In *S*. *littoralis*, pre-exposure experiments and behavioural pheromone tests were performed only with sexually mature 2- to 3-day-old males and it was suggested that pre-exposure elicits a sensitization to the pheromone by enhancing peripheral and central neuron sensitivity [25, 26, 28, 39]. Here we show that the behavioural pheromone pre-exposure effect is age-independent in *A*. *ipsilon*: it was revealed in both mature males and young immature males, indicating that pre-exposure might accelerate maturation of the pheromone processing system, possibly by enhancing the neuron sensitivity in the AL. Similar acceleration of olfactory maturation resulting in an increase of behavioural responses to pheromone was obtained after treatments of sexually immature males with the two main insect hormones, juvenile hormone (JH) and 20-hydroxyecdysone (20E) [29, 30, 38]. Whether pre-exposure could enhance the antennal and/or AL sensitivity via an increase of JH or 20E biosynthetic activity remains to be tested.

As opposed to the age-independent pre-exposure effects, we did not observe a behavioural effect of oral treatment with 10 ng of clothianidin in young immature males, a dose which has previously been shown to elicit an hormetic-like effect, *i*.*e*. increased pheromone responses in sexually mature males [12]. Even though this result shows that clothianidin effects seem to be age-dependent in *A*. *ipsilon*, further studies using different doses of the insecticide are needed to confirm this result. Our toxicity tests of clothianidin on *A*. *ipsilon* have shown that young males are less affected by the same doses than older males (data not shown), whereas the dose of 10 ng corresponds to the LD20 in 5-day-old males, it does not cause any mortality in 2-day-old males, which could be due to the differences in general physiology between the ages, as mentioned above. A differential effect of sublethal doses of insecticides as a function of age has been described in other insects, and might be due among others to varying activity of detoxification enzymes at different age [40–42]. Indeed, the over-expression of enzymes such as cytochrome P450 is associated with high resistance to neonicotinoids in many insect species [43], and the age-specific expression of a P450 monooxygenase was shown to correlate with neonicotinoid resistance in the white fly [44].

When combining pre-exposure and clothianidin treatments in mature males, we found an additive effect on behavioural responses, which indicates that sensory experience influences the effect of the insecticide in this target insect. These combined effects lead to the question, if experience and insecticide effects (and eventually also age-effects) might originate from similar modulatory mechanisms.

Even though the olfactory system of *S*. *littoralis* is modified by brief pre-exposure, eliciting increased sensitivity of peripheral and more pronouncedly in AL neurons as well as an increase in the volume of the pheromone-processing cumulus of the macroglomerular complex within the antennal lobe and the calyces of the secondary olfactory centres, the mushroom bodies [25, 27, 28], nothing is known so far on the neuromodulators potentially involved in these changes.

In *A*. *ipsilon*, on the other hand, we have previously shown that the maturation of behavioural and neuronal pheromone responses in *A*. *ipsilon* can be accelerated by hormonal treatments, *i*.*e*. injections of JH mimetics or ecdysone [29, 30, 38], at the behavioural level by the application of dopamine [45] and at the neuronal level, without behavioural effect, by application of octopamine [36]. In addition to the effects on immature males, we show here that pre-exposure of sexually mature males still enhances their behavioural pheromone response, reaching very high levels (more than 80% responses). Moreover, combination of pre-exposure and clothianidin induced even higher pheromone responses (90%) in males for which, in addition, the proportion of landings was very high. Lastly the delay of response following either pre-exposure, clothianidin treatment or the combination of the two was very short, indicating that the tuning and processing of the sex pheromone components within the AL occurred very rapidly for these treated males. A similar effect (high percentage and fast response) was obtained after dopamine treatments [45]. These results further confirm that behavioural responses of naive sexually mature *A*. *ipsilon* males can still be increased by neuromodulation, as previously shown for example by octopamine treatments [36], but not ecdysone treatments [38].

As for neuronal plasticity as a function of age, the increased behavioural responses of *A*. *ipsilon* males to the pheromone after a treatment with 10 ng of clothianidin are correlated with increased sensitivity of AL neurons [11]. We can therefore speculate that similar modulatory mechanisms might be involved in the two processes. In support of this hypothesis, an increase in dopamine synthesis has been reported in the rat brain after clothianidin treatment [46]. Future studies should therefore explore the role of this biogenic amine in the different forms of olfactory plasticity, including insecticide effects.

The behavioural increase of sex pheromone responses after clothianidin treatment has previously been shown to be linked with increased sensitivity of central olfactory neurons [11]. This sensitivity change could be due to the presence of specific nAchR subunits in inhibitory neurons of the olfactory pathway, which might desensitize through clothianidin treatment [47, 48]. Alternatively clothianidin might indirectly act via modulation of dopamine levels in the brain ([11] and references therein).

## Conclusions

We present here *A*. *ipsilon* as a favorable model insect to study neural and molecular mechanisms of sensory plasticity including effects caused by insecticides, because broad knowledge on different forms of plasticity is accumulating in this insect with an easily accessible nervous system, available transcriptomic information and well-established behavioural tests. We might now be able to link behavioural effects with molecular and cellular mechanisms by combining molecular, pharmacological and electrophysiological approaches. Understanding these mechanisms will help to subsequently design further experiments investigating the ecological impact of neonicotinoid insecticides.

In addition, our findings provide important information in the context of plant protection, because they show that an insecticide can, via the bias of their sensory systems, increase reproduction of a pest insect even further as a function of behavioural and neural plasticity. When studying effects of sublethal doses of insecticides, plasticity of sensory systems thus has to be taken into account.
